# Everolimus-Eluting Stent (Espalier) in Coronary Artery Revascularization: A Retrospective, Investigator-Initiated, Multicenter Study

**DOI:** 10.14740/cr2200

**Published:** 2026-06-05

**Authors:** Parminder Singh Manghera, Gaurav Mohan, Amit Kumar, Harpreet Singh Gilhotra, Sushil Sharma, Deepti Goel, Roma Tiwari

**Affiliations:** a Department of Cardiology, Government Medical College, Amritsar, Punjab 143001, India; b Department of Medicine, Sri Guru Ram Das (SGRD) Institute of Medical Sciences and Research, Amritsar, Punjab 143501, India; c Department of Cardiology, Indus Super Speciality Hospital, Sahibzada Ajit Singh Nagar, Mohali, Punjab 160062, India; d Sohana Hospital, Sahibzada Ajit Singh Nagar, Punjab 140308, India; e Cognitive Health Technologies Ltd., New Delhi, India; f Harrison’s Tech Consultants, Mumbai, India; g Clinical Operations, Harrison’s Tech Consultants, Mumbai, India

**Keywords:** PCI, Stent, CAD, Drug-eluting stent, STEMI

## Abstract

**Background:**

Drug-eluting stents (DESs) are key to percutaneous coronary intervention (PCI), with outcomes shaped by device, patient, and operator factors. The everolimus-eluting stent (EES, Espalier) was assessed in a multicenter, real-world study for safety and efficacy. The study evaluated the clinical outcomes, safety, and procedural success of the indigenous Espalier EES (Cognitive Technologies Pvt. Ltd.) in patients undergoing coronary revascularization at tertiary care centers in India.

**Methods:**

This retrospective, multicenter study enrolled 402 adults (≥ 18 years) with native coronary lesions suitable for PCI. The primary endpoint was 12-month target lesion failure (TLF: cardiac death, target vessel myocardial infarction (MI), emergent coronary artery bypass grafting (CABG), or clinically driven target lesion revascularization (TLR)). Secondary endpoints included 6-month TLF, target vessel revascularization (TVR), stent thrombosis, and device/procedural success. Subgroup analyses were performed by age, sex, body mass index (BMI), vessel size, and clinical presentation.

**Results:**

At 6 months, adverse events were minimal (0.50%), with no MI or stent thrombosis, one death (0.25%), and one TLF (0.25%). At 12 months, mortality remained 0.25%, with no MI or stent thrombosis, while TVR occurred in 1.49% and TLF in 1.74% of patients. Subgroup analyses revealed higher event rates in older patients (≥ 70 years), those with unstable angina, abnormal electrocardiogram (ECG)/echocardiographic findings, and patients with two comorbidities. Device and procedural success were achieved in all cases (100%), with no failures recorded.

**Conclusions:**

The Espalier EES demonstrated high procedural success and low adverse event rates at 12 months, with slightly higher risks observed in elderly and high-risk patients.

## Introduction

Coronary artery disease (CAD) is a condition defined by the progressive buildup of atherosclerotic plaques within the coronary arteries, which may remain asymptomatic in its early stages. Epidemiological data suggest that men are more susceptible to developing CAD compared to women [1–3]. Coronary revascularization is a key therapeutic approach in managing CAD, with percutaneous coronary intervention (PCI) being widely adopted. PCI involves placing expandable metallic stents into atherosclerotic, stenotic coronary segments to restore and maintain adequate blood flow [4, 5].

Drug-eluting stents (DESs) are tiny mesh tubes placed in heart arteries to keep them open. They release medicine that helps prevent the artery from narrowing again due to plaque buildup [6]. DES is made up of three essential parts: the metal scaffold, a therapeutic drug, and a delivery matrix. The scaffold, most often constructed from stainless steel or cobalt–chromium, provides durable structural support to prevent the vessel from collapsing. The drug component interferes with intracellular signaling and cell cycle activity at different stages, which limits smooth muscle cell growth and reduces the risk of neointimal hyperplasia at the site of implantation [4]. Everolimus prevents coronary restenosis through localized drug release that inhibits cell growth and supports endothelial recovery. Everolimus-eluting stents (EESs) are designed to reduce coronary artery restenosis by releasing everolimus, a drug that prevents cell proliferation and supports vascular healing [7]. The appropriateness of real-world stenting continues to be a topic of discussion [6].

Espalier is an indigenous EES made in India (Cognitive Health Technologies Pvt. Ltd.). Everolimus is an effective immunosuppressant and one of the most frequently used and well-established drugs in the “limus” group. Espalier is designed to deliver everolimus through an optimum blend of biodegradable polymers, enabling controlled drug elution that effectively reduces restenosis [8].

This retrospective study evaluated real-world data by collecting and analyzing clinical outcomes in patients who received EES (Espalier) during PCI, along with dual antiplatelet therapy (DAPT), within an Indian context. The study was specifically designed to gather clinical evidence regarding the performance and safety of the stent in patients treated at four centers across India during 2022 and 2023, reflecting typical daily clinical practice.

## Materials and Methods

### Study design and population

This retrospective, multicentric, study analyzed the clinical performance of the Espalier EES in patients undergoing coronary revascularization across four tertiary care centers in India. Eligible participants were adults aged 18 years or older, of either gender, presenting with single- or multi-vessel lesions in native coronary arteries suitable for angioplasty. Clinical indications included unstable angina (UA), stable ischemic heart disease (SIHD), and non-ST elevation myocardial infarction (NSTEMI). Inclusion required complete medical records encompassing baseline, post-procedure, and follow-up data at 6 and 12 months. Data of patients who received the stent and received DAPT post-coronary artery bypass grafting (CABG) were included. Diabetic patients were enrolled if previously diagnosed and on medication, or if they met American Diabetes Association (ADA) criteria based on glycated hemoglobin (HbA1c), fasting plasma glucose, or 2-h glucose levels. Exclusion criteria comprised hemodynamic instability or cardiogenic shock, contraindications to DAPT, chronic total occlusion or aorto-ostial lesions, prior CABG, vessel diameters < 2.5 mm or > 4 mm, and lesions with in-stent restenosis. This inclusive, real-world design enabled a comprehensive evaluation of the stent’s safety and performance across a diverse patient population. All eligible patients identified from records were included in the analysis. No selection based on lesion complexity, operator preference, or procedural circumstances was applied. Procedural success was defined as successful delivery and deployment of the stent at the target lesion with acceptable angiographic outcome. Clinical follow-up was performed at 30 days, 6 months, and 12 months using outpatient visits, hospital record review, and/or telephonic contact.

### Ethical considerations

The research adhered to both national and international ethical guidelines, such as the New Drugs and Clinical Trials Rules, 2019 (Central Drugs Standard Control Organization (CDSCO), India; Approved No. (MFG/MD/2023/000595)), the Indian Council of Medical Research (ICMR) Ethical Guidelines for Biomedical Research (2017), the International Council for Harmonisation (ICH) E6 (R3) Good Clinical Practice (GCP), and the Declaration of Helsinki (48th General Assembly). Approvals from the ethics committee were secured prior to starting, and the study began within 5 months of regulatory clearance. Data were collected retrospectively from institutional records detailing baseline information, procedures, and follow-ups at 6 and 12 months. All participating sites maintained regulatory compliance, prioritized participant safety, and upheld ethical standards throughout the duration of the study.

### Study objectives and outcomes

The clinical outcomes in this study were evaluated based on both primary and secondary objectives. The primary objective involved assessing target lesion failure (TLF) at 12 months. TLF was defined as a composite endpoint including cardiac mortality, target vessel myocardial infarction (MI, Q-wave or non-Q-wave), emergent CABG, and clinically driven target lesion revascularization (TLR). TLR referred to a repeat procedure, either percutaneous or surgical, undertaken to treat a previously stented segment of a coronary artery following restenosis.

Secondary objectives included TLF at 6-month follow-up, clinically driven target vessel revascularization (TVR) and TLR at both 6 and 12 months, stent thrombosis rate as per the Academic Research Consortium (ARC) definition, clinical device success (successful delivery and deployment of the stent), and procedural success (achievement of < 30% residual stenosis without major adverse cardiac events (MACEs) during hospitalization). Additionally, a structured schedule of events and data collection was implemented to ensure consistent follow-up and outcome assessment. Assessment of device safety was conducted through analysis of adverse device effects (ADEs) and serious adverse device effects (SADEs).

### Device description

Espalier is an indigenous EES developed in India by Cognitive Health Technologies Pvt. Ltd. Everolimus inhibits growth factor–driven cell proliferation by forming a complex with FKBP-12, which disrupts mTOR signaling and arrests the cell cycle at the late G1 phase, thereby suppressing cellular metabolism and proliferation. The stent releases 50–280 µg of everolimus (1.0 µg/mm^2^) over 45 days, with 60% eluted within the first 15 days and the remaining 40% gradually released, ensuring restenosis prevention while supporting endothelial healing.

### Procedural and post-PCI medications

DAPT, comprising agents such as clopidogrel (≥ 75 mg daily), ticlopidine (250 mg twice daily), prasugrel (10 mg), or ticagrelor (90 mg), was recommended for patients undergoing PCI. In cases of acute coronary syndrome (ACS), DAPT was advised for a duration of 12 months, whereas in elective PCI, it was prescribed for at least 6 months or as per the prevailing local standard of care.

### Sample size

The study aimed to collect data from 500 patients based on feasibility and statistical needs. As a retrospective study, the final sample depended on available patient records at participating centers. Ultimately, 402 records met the inclusion criteria and were included in the analysis. Enrollment was not extended beyond this period to maintain consistency in follow-up duration and data completeness.

### Statistical analysis

As this was an observational study involving retrospective data analysis, no study hypothesis was formulated and no formal comparisons were performed. However, subgroup analyses were done. Measurement data for demography (age, height, weight, body mass index (BMI), and vital parameters) were analyzed for differences between the subgroups based on age, gender and vessel size, using one way analysis of variance (ANOVA). All analyses were done using two-sided tests at alpha 0.05 (95% confidence levels).

Analyses of both primary and secondary endpoints were conducted on the total subject cohort, representing the intention-to-treat (ITT) dataset. Pre-specified subgroup analyses were also performed, including patients with small vessels (n = 70, target vessel diameter of ≤ 2.75 mm and > 2.75 mm), and MI (ST-elevation myocardial infarction (STEMI) and NSTEMI) as per New York Heart Association (NYHA) criteria at presentation (n = 100). Additional subgroup evaluations were carried out based on gender and age groups (≤ 40 years, 40–50 years, 50–60 years, 60–70 years, and ≥ 70 years).

## Results

### Baseline demographics

Table 1 present the study population, which included 402 patients, with a male predominance (59.9%). Most were either overweight (48.0%) or normal BMI (32.6%), while obesity was present in 18.9%. Most patients were between 50 and 70 years of age (64.7%), with 20.6% aged ≥ 70 years and only 4% younger than 40 years. Clinical presentation was dominated by STEMI (65.2%), followed by UA (18.7%) and NSTEMI (14.9%), whereas stable angina accounted for just 1.2%. Single- or two-vessel disease was most common (76.9%), with multivessel involvement beyond three vessels being infrequent. Pre-procedural abnormalities were noted in 95.3% of electrocardiograms (ECGs) and 55.5% of echocardiograms. Regarding comorbidities, 42.8% had one medical condition, 32.6% had two, 8.5% had three, while 16.2% had no prior history.

**Table 1:** Profile of Patients (N = 402)

	N	%
Sex^a^		
Male	241	59.95%
Female	161	40.05%
Age group^b^		
≤ 40 years	16	3.98%
40–50 years	43	10.70%
50–60 years	117	29.10%
60–70 years	143	35.57%
≥ 70 years	83	20.65%
BMI category^c^		
Underweight	2	0.50%
Normal	131	32.59%
Overweight	193	48.01%
Obese	76	18.91%
Vessel size^d^		
Not small vessel	302	75.12%
Small vessel (≤ 2.75 mm)	100	24.88%
Medical history		
No medical history	65	16.17%
One medical condition	172	42.79%
Two medical conditions	131	32.59%
Three medical conditions	34	8.46%
Medical condition		
Dyslipidemia	10	2.49%
Hypertension	258	64.18%
Family history of CAD	1	0.25%
Previous PCI	24	5.97%
Previous CABG	1	0.25%
Renal dysfunction	2	0.50%
Stroke	3	0.75%
Asthma/COPD	7	1.74%
Smoking	26	6.47%
Alcohol	28	6.97%
Diabetes mellitus	176	43.78%
Concomitant medication	321	79.85%
Indication for PCI		
Stable angina	5	1.24%
NSTEMI	60	14.93%
STEMI	262	65.17%
Unstable angina	75	18.66%
Other indications for PCI		
Stable angina	5	1.24%
NSTEMI	57	14.18%
STEMI	258	64.18%
Unstable angina	84	20.90%
Asymptomatic (positive stress test)	2	0.50%
Others	2	0.50%
ECG (pre-PCI)^c^		
Normal	19	4.73%
Abnormal	383	95.27%
Echo (pre-PCI)		
Normal	179	44.53%
Abnormal	223	55.47%

^a^Sex refers to the biological classification of participants. ^b^Age group: stratification of participants. ^c^BMI category: classification based on BMI. ^d^Vessel size: coronary artery diameter. ^c^ECG (pre-PCI): ECG findings before PCI. BMI: body mass index; CAD: coronary artery disease; MI: myocardial infarction; PCI: percutaneous coronary intervention; STEMI: ST-elevation myocardial infarction; NSTEMI: non-ST elevation myocardial infarction; ECG: electrocardiogram; CABG: coronary artery bypass graft; COPD: chronic obstructive pulmonary disease.

### Clinical outcomes at 6 and 12 months (n = 402)

At 6 months, adverse events were minimal (0.50%), with no cases of MI or stent thrombosis reported. Mortality was observed in only one patient (0.25%), while TVR was not required. TLF occurred in a single patient (0.25%). At 12 months, outcomes remained favorable, with no MI or stent thrombosis recorded. Mortality remained unchanged at 0.25%. However, six patients (1.49%) required TVR, and seven patients (1.74%) experienced TLF. Overall, the Espalier EES demonstrated sustained safety and low adverse event rates over the 12-month follow-up period (Table 2).

**Table 2:** Clinical Outcomes at 6 and 12 Months (N = 402)

	6 months	12 months
MI, n (%)	0 (–)	0 (–)
Death^a^, n (%)	1 (0.25%)	1 (0.25%)
Stent thrombosis^b^, n (%)	0 (–)	0 (–)
TVR, n (%)	0 (–)	6 (1.49%)
TLF^c^, n (%)	1 (0.25%)	7 (1.74%)

^a^Death: number and percentage of patients who died during the follow-up period. ^b^Stent thrombosis: number and percentage of cases where a blood clot formed in the implanted stent. ^c^TLF: composite of cardiac death, target vessel Q-wave or non-Q wave MI, emergent coronary artery bypass graft (CABG), clinically driven target lesion. MI: myocardial infarction; TVR: target vessel revascularization; TLF: target lesion failure.

### Clinical outcomes at 12 months in different subgroups

At 12 months, overall adverse event rates remained low across subgroups. Among sexes, males experienced four cases of TVR and TLF (1.66%), while females had slightly higher mortality (0.62%) and three cases of TLF (1.86%). By BMI, normal-weight patients showed the highest TLF incidence (3.05%), whereas overweight and obese patients had lower rates (1.04% and 1.32%, respectively), and no events occurred in underweight individuals. Age stratification revealed increasing event rates with advancing age: patients ≥ 70 years had the highest burden (death 1.20%, TVR 2.41%, TLF 3.61%), while younger groups (< 40 years) had no adverse outcomes. Regarding PCI indication, STEMI patients accounted for most events (death 0.38%, TVR 1.15%, TLF 1.53%), whereas UA was associated with the highest TVR/TLF rates (4.0% each). Patients with single-vessel disease had slightly higher mortality (0.66%) and TLF (2.65%) compared to those with multivessel involvement, where rates were lower. Pre-procedural ECG abnormalities were linked to most events (death 0.26%, TVR 1.57%, TLF 1.83%), while patients with normal ECG had none. Similarly, abnormal echocardiographic findings were associated with higher event rates (death 0.45%, TLF 2.24%) compared to normal echocardiographic findings (1.12%). Finally, comorbidity burden influenced outcomes: patients with two medical conditions had the highest TLF (3.05%), while those with no or three conditions had no adverse events (Table 3).

**Table 3:** Clinical Outcomes at 12 Months in Different Subgroups

	N	Death^a^	TVR	TLF^b^
Sex				
Male	241	0 (–)	4 (1.66%)	4 (1.66%)
Female	161	1 (0.62%)	2 (1.24%)	3 (1.86%)
BMI category				
Underweight	2	0 (–)	0 (–)	0 (–)
Normal	131	0 (–)	4 (3.05%)	4 (3.05%)
Overweight	193	1 (0.52%)	1 (0.52%)	2 (1.04%)
Obese	76	0 (–)	1 (1.32%)	1 (1.32%)
Age group				
≤ 40 years	16	0 (–)	0 (–)	0 (–)
40–50 years	43	0 (–)	1 (2.33%)	1 (2.33%)
50–60 years	117	0 (–)	1 (0.85%)	1 (0.85%)
60–70 years	143	0 (–)	2 (1.40%)	2 (1.40%)
≥ 70 years	83	1 (1.20%)	2 (2.41%)	3 (3.61%)
Indication for PCI				
Stable angina	5	0 (–)	0 (–)	0 (–)
NSTEMI	60	0 (–)	0 (–)	0 (–)
STEMI	262	1 (0.38%)	3 (1.15%)	4 (1.53%)
Unstable angina	75	0 (–)	3 (4.00%)	3 (4.00%)
Number of vessels assessed				
One vessel	151	1 (0.66%)	3 (1.99%)	4 (2.65%)
Two vessels	158	0 (–)	1 (0.63%)	1 (0.63%)
Three vessels	78	0 (–)	2 (2.56%)	2 (2.56%)
Four vessels	14	0 (–)	0 (–)	0 (–)
Five vessels	1	0 (–)	0 (–)	0 (–)
ECG (pre-PCI)				
Normal	19	0 (–)	0 (–)	0 (–)
Abnormal	383	1 (0.26%)	6 (1.57%)	7 (1.83%)
Echo (pre-PCI)				
Normal	179	0 (–)	2 (1.12%)	2 (1.12%)
Abnormal	223	1 (0.45%)	4 (1.79%)	5 (2.24%)
Medical history				
No medical history	65	0 (–)	0 (–)	0 (–)
One medical condition	172	1 (0.58%)	2 (1.16%)	3 (1.74%)
Two medical conditions	131	0 (–)	4 (3.05%)	4 (3.05%)
Three medical conditions	34	0 (–)	0 (–)	0 (–)

There were no incidences of MI and stent thrombosis. ^a^Death: number and percentage of patients who died during the follow-up period. ^b^TLF: composite of cardiac death, target vessel Q-wave or non-Q wave MI, emergent coronary artery bypass graft (CABG), clinically driven target lesion. MI: myocardial infarction; TVR: target vessel revascularization; PCI: percutaneous coronary intervention; STEMI: ST-elevation myocardial infarction; NSTEMI: non-ST elevation myocardial infarction; ECG: electrocardiogram; TLF: target lesion failure.

Table 4 shows the Espalier compliance chart.

**Table 4:** Espalier Compliance Chart

Pressure (bar)	Diameter (mm)							
	2.00	2.25	2.50	2.75	3.00	3.50	4.00	4.50
6	1.920	2.116	2.352	2.580	2.802	3.252	3.852	4.417
7	1.941	2.160	2.402	2.640	2.901	3.302	3.902	4.436
8	1.961	2.211	2.461	2.710	2.943	3.401	3.951	4.451
9	2.007^a^	2.252^a^	2.504^a^	2.752^a^	3.004^a^	3.503^a^	4.004^a^	4.502^a^
10	2.043	2.340	2.602	2.811	3.072	3.542	4.042	4.521
11	2.084	2.382	2.643	2.864	3.101	3.582	4.082	4.552
12	2.102	2.422	2.682	2.917	3.143	3.622	4.122	4.584
13	2.130	2.440	2.703	2.950	3.184	3.662	4.161	4.603
14	2.160	2.460	2.722	2.983	3.203	3.701	4.203	4.623^b^
15	2.180	2.480	2.743	3.012	3.222	3.743	4.241	4.640
16	2.200^b^	2.503^b^	2.762^b^	3.047^b^	3.242^b^	3.782^b^	4.282^b^	4.674
17	2.222	2.541	2.802	3.072	3.273	3.822	4.322	4.710
18	2.241	2.562	2.823	3.103	3.350	3.861	4.363	4.751

^a^Nominal pressure (NP). ^b^Rated burst pressure (RBP).

### Clinical device and procedural success

Clinical device success was achieved in all cases (100%), with no instances of failure recorded.

## Discussion

Several Indian studies have investigated the safety and efficacy of EESs in patients undergoing PCI for CAD, contributing to a growing body of evidence supporting their use in diverse clinical settings [9, 10]. These studies have consistently demonstrated favorable outcomes with EES in terms of TLR, stent thrombosis rates, and long-term MACEs.

In this multicentric, retrospective study of 402 patients undergoing PCI with the indigenous Espalier EES across four tertiary centers in India, device and procedural success was achieved in all cases (100%). At 12 months, no stent thrombosis or periprocedural MI was reported. Mortality was 0.25%, while TVR and TLF occurred in 1.49% and 1.74% of patients, respectively. These results highlight the procedural reliability, technical deliverability, and long-term safety of the Espalier EES, aligning favorably with international benchmarks and underscoring its effectiveness in real-world Indian practice.

Comparable studies of thin-strut EES have shown similarly favorable outcomes in a Korean multicenter ACS cohort (n = 922). The SYNERGY™ biodegradable polymer EES demonstrated safety and efficacy, with low 3-year event rates of patient-oriented composite outcome (POCO) of 9.9%, device-oriented composite outcome (DOCO) of 3.6%, and minimal stent thrombosis (0.3%). The study population is more comparable to contemporary observational studies in ACS, such as that reported by Sim et al [11]; however, the multicenter retrospective nature of our study likely reflects greater heterogeneity in patient and lesion characteristics. For instance, the study by Stone et al (2010) [8] which evaluated the Xience V EES in over 3,600 patients, reported a 1-year TLF rate of 4.2% and stent thrombosis rate of 0.29%. In comparison to the SPIRIT IV trial, which predominantly included patients with stable CAD in a highly controlled setting, the present study represents a more real-world cohort with a predominance of ACS patients. The present findings should be interpreted in the context of outcomes reported with contemporary EESs such as XIENCE and SYNERGY. These large-scale studies typically included more heterogeneous patient populations with a greater proportion of stable CAD, whereas the present cohort was predominantly composed of ACS patients, reflecting real-world tertiary care practice. Despite this higher-risk clinical profile, the rates of TLF and TLR observed in our study are broadly consistent with those reported for XIENCE and SYNERGY platforms. This suggests that the performance of the study stent is comparable to other second-generation limus-eluting stents in routine clinical use. Similarly, the COMPARE trial comparing everolimus vs. paclitaxel DESs showed a 1-year TLF of 5.2% in the everolimus group [12]. The performance of Espalier stent in a real-world, high-risk cohort with high rates of STEMI (65.17%), hypertension (64.18%), and diabetes (43.78%) demonstrates its clinical robustness. Likewise, a pooled analysis of 19 randomized controlled trials (RCTs) and two registries (103,101 patients) showed that second-generation ultrathin strut stents reduced TLF at 1, 2, and 3 years, but no benefit was observed at 5 years. Secondary outcomes (TLR, TVR, targets vessel myocardial infarction (TVMI)) followed the same pattern [13]. These findings align with our results, suggesting that thinner strut design and optimized polymer technology contribute to improved endothelialization, reduced vessel injury, and lower risk of restenosis or thrombosis [14].

Pre-procedural cardiac assessments revealed ECG abnormalities in 95.27% of patients and echocardiographic abnormalities in 55.47%, consistent with ACS presentations. These findings underscore the high disease burden and justify the need for effective stent platforms. The left anterior descending (LAD) coronary artery and right coronary artery (RCA) were the most frequently treated vessels, mirroring global PCI trends.

The study also explored anatomical and procedural metrics across age, sex, and vessel size. While most parameters showed no variation, stent diameter differed by age, and small vessels (≤ 2.75 mm) required shorter and narrower stents, confirming tailored procedural strategies. These insights are consistent with findings from the RESOLUTE All-Comers trial by Iqbal et al (2015), which emphasized the importance of vessel size in stent selection and outcomes [15].

Importantly, the Espalier DES demonstrated zero device failure and uniform success across lesion types, including complex multi-stent deployments. This supports its adaptability and reliability in diverse anatomical scenarios. The absence of sex-based differences in stent metrics, despite higher variability among males, suggests procedural heterogeneity rather than anatomical disparity.

Indigenous stent platforms have also shown encouraging safety profiles in Indian cohorts. A postmarketing study of FlexyRap biodegradable polymer DES reported 100% device success and a 6.25% MACE rate at 24 months [16]. Similarly, real-world data on indigenous biodegradable polymer DES demonstrated low rates of stent thrombosis and revascularization, supporting their safety and efficacy in routine practice. Compared with these, the Espalier DES in our study achieved even lower adverse event rates, highlighting its robustness and reliability [17]. The single death observed occurred in a high-risk patient subgroup, an elderly individual with multiple comorbidities and abnormal baseline cardiac findings. The patient underwent stent placement on June 23, 2023, and subsequently died on November 16, 2023. She was an 83-year-old woman with breast carcinoma, status post surgery, chemotherapy, and radiotherapy, with documented progressive disease. As per the decision of the patient and her family, all further cancer-directed treatment had been discontinued. The interval between stent placement and death does not suggest any temporal or causal association with the device. In the absence of stent thrombosis, MI, or procedural complications, the event is unlikely to be directly attributable to the stent; however, causality cannot be definitively established given the retrospective design. The death is most plausibly explained by her advanced age, comorbidity burden, progressive malignancy, and underlying cardiac conditions. She passed away peacefully at home, and the family declined an autopsy.

This favorable clinical outcomes also highlight the optimal technical execution and reliability of the interventional equipment, underscoring both procedural efficacy and safety across the study population. The absence of procedural failure further reflects high operator proficiency, effective device deployment, and appropriate patient selection. Such uniform success emphasizes the procedural reliability and safety of PCI in this cohort. Subgroup analyses presented in this study are descriptive in nature and should be interpreted with caution, as the study was not powered to detect statistically significant differences between groups. These observations are intended to provide an exploratory overview of event distribution across clinically relevant patient subgroups in a real-world setting.

The strengths of the study include its multicentric design, a relatively large cohort of 402 patients across four tertiary care centers, employing an inclusive real-world design that encompassed diverse clinical presentations such as STEMI, NSTEMI, UA, and SIHD. Rigorous follow-up at 6 and 12 months demonstrated complete device success, while subgroup analyses consistently confirmed the safety profile across sex, BMI categories, age groups, extent of vessel involvement, and varying comorbidity burdens. The observed 100% procedural success rate should be interpreted in the context of the study definition (successful stent delivery and deployment) and the retrospective design. Although consecutive patients were included, the possibility of unmeasured selection bias inherent to retrospective analyses cannot be entirely excluded.

### Limitations

Limitations include its retrospective nature, lack of a control group, absence of angiographic follow-up, short-term secondary endpoint evaluation, and geographic concentration in North India. Additionally, although the study included 402 patients across multiple centers, the sample size remains relatively modest for drawing definitive conclusions, and larger prospective studies are warranted to further validate these findings. The low proportion of patients with stable angina in this study likely reflects the case-mix at participating tertiary care centers, which predominantly manage ACS cases. As a result, patients with stable CAD may be underrepresented, which may limit the generalizability of the findings to the broader PCI population. While baseline ECG and echocardiographic findings were available, these may not fully reflect lesion-level disease complexity. More detailed procedural and anatomical characteristics, such as lesion calcification, use of atherectomy or intravascular lithotripsy, lesion diffuseness, and bifurcation involvement, were not consistently captured due to the retrospective design and represent an important limitation of the study. Despite these limitations, the study provides compelling evidence supporting the clinical utility of the Espalier DES in diverse anatomical and demographic settings.

### Conclusions

This multicentric, retrospective study demonstrated the favorable clinical performance and safety of the indigenous Espalier EES in patients undergoing PCI for CAD. The primary endpoint 12-month TLF was low at 1.74%, with no cases of MI or stent thrombosis, and only one cardiac death (0.25%), indicating long-term procedural outcomes. Secondary endpoints further reinforced these findings, with a 6-month TLF rate of just 0.25%, favorable safety and performance of the Espalier EES, and minimal early complications. Overall, the study supports the efficacy, safety, and adaptability of the Espalier EES across diverse patient profiles and clinical settings. The impact on daily practice is that the Espalier EES ensures favorable clinical outcomes with low rates of adverse events, offering a safe, reliable, and cost-effective option for PCI in diverse real-world patients, thereby enhancing accessibility and confidence in indigenous stent use.

## Data Availability

The data supporting the findings of this study are available from the corresponding author upon reasonable request.

## Abbreviations

CAD: coronary artery disease
DES: drug-eluting stent
EES: everolimus-eluting stent
MACEs: major adverse cardiac events
MI: myocardial infarction
PCI: percutaneous coronary intervention
SES: sirolimus-eluting stent
STEMI: ST-elevation myocardial infarction
TIMI: thrombolysis in myocardial infarction
TLR: target lesion revascularization
TVMI: targets vessel myocardial infarction
TVR: target vessel revascularization
